# Mixing rate in Classical Many Body Systems

**DOI:** 10.1038/s41598-019-47269-3

**Published:** 2019-09-04

**Authors:** Gad Frenkel, Moshe Schwartz

**Affiliations:** 1 Faculty of Engineering, Ruppin Academic Center, Emek-Hefer, 40250 Monash, Israel; 2 School of Physics and Astronomy, Raymond and Beverly Faculty of Exact Sciences, Tel Aviv University, 69978 Tel Aviv, Israel

**Keywords:** Structure of solids and liquids, Structure of solids and liquids, Glasses, Statistical physics, Phase transitions and critical phenomena

## Abstract

Mixing in many body systems is intuitively understood as the change in time of the set of neighbors surrounding each particle. Its rate and its development over time hold important clues to the behavior of many body systems. For example, gas particles constantly change their position and surrounding particles, while in solids one expects the motion of the atoms to be limited by a fixed set of neighboring atoms. In other systems the situation is less clear. For example, agitated granular systems may behave like a fluid, a solid or glass, depending on various parameter such as density and friction. Thus, we introduce a parameter which describes the mixing rate in many body systems in terms of changes of a properly chosen adjacency matrix. The parameter is easily measurable in simulations but not in experiment. To demonstrate an application of the concept, we simulate a many body system, with particles interacting via a two-body potential and calculate the mixing rate as a function of time and volume fraction. The time dependence of the mixing rate clearly indicates the onset of crystallization

## Introduction

Many body systems exhibit a myriad of phases, which may be stable or metastable. The elementary classification of those phases is into fluid phases where no spatial order exists and frozen phases. The fluid phases may be stable, like gases or ordinary liquids, or metastable like super cooled liquids. Frozen systems include periodic ordinary stable solids, but also metastable periodic solids like superheated solids. We encounter, in addition and rather mundanely, amorphous solids, which are entirely disordered. Glasses form excellent examples, regardless of the question if glass is a thermodynamic phase or not^1–3^. Another set of examples is offered by granular aggregates, that exhibit amorphous solid structure, after cooling^4–11^ and remain in a liquid like phase if constantly vibrated^12–20^. Of particular recent interest is the phenomenon of jamming under shear in which the transition from the liquid to the glassy state is affected by external shear^21–28^.

When speaking about frozen systems a naïve picture springs to mind in which the position of each particle is fixed relatively to the other particles, in contrast to particles in a fluid which keep changing their relative positions. In some cases, like in granular systems after the kinetic energy in the center of mass system has been spent, this picture is correct. In other systems, like ordered solids or glasses, the relative positions of particles keep changing all the time, due to finite temperature. (Even at very low temperature the particles cannot be seen as fixed due to zero-point motion, but in that case the system is not in the classical regime.) Therefore, the naïve criterion described above, when taken strictly is not really helpful. It is true, though, that in a frozen system, each particle sees for long times, more or less, the same neighbors. This observation serves now as a basis for defining the parameter of mixing rate, that may distinguish in a continuous manner between less frozen and more frozen systems.

Our starting point is a microscopic length, *l*, relevant to the system. This length can be chosen in many ways. It can be given by *l* = *αρ*^−1/3^, where *α* > 1 but is still of order 1 and *ρ* is the number density of particles in the system. Alternatively, it can be chosen as a typical range of the interaction etc. Once *l* is given we define an adjacency matrix, **A**, which gives the neighborhood relation between particles in the system,

1 $${{\bf{A}}}_{ij}=\{\begin{array}{c}1\,{\rm{if}}\,|{{\bf{r}}}_{i}-{{\bf{r}}}_{j}| < l\\ 0\,{\rm{otherwise}}\end{array},$$

where **r**_*i*_ is the position vector of particle *i*. Since the position of each particle is time dependent, so is the adjacency matrix. Our purpose is to define a single parameter, which will describe the change of the adjacency matrix in time. Now suppose we have the adjacency matrix at time *t*, and at a later time *t* + Δ*t*. (The time Δ*t* can be chosen as the number of elementary steps needed in a simulation to update the positions of all particles or a multiple of that number that will ensure that some small but sizeable change in the adjacency matrix will be observed.) We define the Hamming distance, between those two matrices,

2 $$H(t,\,\Delta t)=\mathop{\sum }\limits_{i,\,j=1}^{N}|{{\bf{A}}}_{ij}(t+\Delta t)-{{\bf{A}}}_{ij}(t)|,$$

where *N* is the total number of particles in the system.

We define next the dimensionless mixing rate,

3 $$f(t,\Delta t)=\frac{H(t,\Delta t)\tau }{N\Delta t}.$$

The number of particles is introduced to take out the size dependence in large systems. The time, *τ*, introduced to render the mixing rate dimensionless, is given by

4 $$\tau =\frac{l}{{v}_{rms}},$$

where *ν*_*rms*_ is the initial root mean square (RMS) velocity of a particle in the center of mass system. The RMS velocity is introduced so that the mixing rate will be less affected by scaling of all the velocities in the system. In systems with conservation of energy we can choose the instantaneous value of the RMS velocity, since eventually the system reaches equilibrium and the RMS velocity is more or less fixed in time. For systems with dissipation the use of the instantaneous value may prove misleading as the dissipation of the kinetic energy might be faster than the rate of decrease of the Hamming distance. Thus, for dissipative systems, the initial RMS velocity will be used, while for systems in which dissipation is accompanied by constant injection of energy (such as vibrated granular systems) the long time steady state RMS velocity should be used.

The selected parameters *l*, *ν*_*rms*_ and the time difference Δt define the scale of the mixing rate. In particular, if Δ*t* is too large, particles may move too far from their initial positions during that time interval. Thus, the Hamming distance will not take into account neighbors encountered in the time interval between *t* and *t* + Δt that left the vicinity defined by *l* before termination of the time interval. The mixing rate will decrease in this case with Δt. To avoid this dependence and measure a significant change in the Hamming distance, $\Delta t\approx l/{v}_{rms}$ should be used.

**Figure 1:**
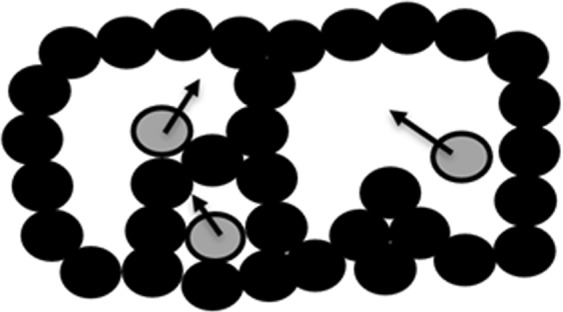
Apparent Mixing. Rattlers (grey) motion in fixed cells induce apparent mixing, as cell sizes are larger than the adjacency length scale *l*.

Equations (3) and (4) describe the basic idea. At first sight, it may seem that the rate defined above does the job. For a frozen system, *f*(*t*, Δ*t*) should vanish, while for a fluid system it should be finite. As we shall see, however, when the system is frozen in the more general sense, the parameter *f* may not be sufficient to determine that the system is not fluid. Consider first an example taken from granular systems. In some cases most of the particles are arranged in solid-like cells (or arches when gravity is involved) and within those cells there may exist rattlers, particles which are confined to the cells but free to move within them. This situation is depicted in Fig. 1. If *l* is about the size of a particle, the only possible neighbors (in the sense of equation (1)) of a rattler are other rattlers in the same cell or particles belonging only to the walls of the same cell. If the cells are larger than *l*, the adjacency matrix will change all the time and the system which we would describe as frozen will have a non-vanishing mixing rate *f*. (If the cell is small enough, the distances between two rattlers in the same cage or distances between a rattler and the wall of the cage remain always smaller than *l*, while distances of a rattler to walls of other cages remain larger than *l*).

While in the figure above most grains stay totally fixed, more general situations, which we would deem frozen, exist, where all particles are in relative motion. In fact, such systems are even more abundant. For example, in a classical crystalline solid, although the neighborhoods are well defined, the adjacency matrix keeps changing due to phonons. The same is true also in glasses, where excitations from the mechanical equilibrium state, present in the system, cause an ever changing adjacency matrix. This would result in a non-vanishing *f*. Consequently, we arrive at the conclusion that our mixing rate defined above should be modified, to cover the case of the generalized frozen state, while keeping the basic idea.

An alternative but related approach is to employ instead of the adjacency matrix at a given time, *t*, a cumulative adjacency matrix that gives not only who are the neighbors at a given time but also the neighbors at earlier and later times.

The cumulative adjacency matrix of order *n* is defined as

5 $${{\bf{A}}}_{n}^{C}(t)=\mathop{\sum }\limits_{i=-n}^{n}\,{}_{\oplus }{\bf{A}}(t+i\Delta t)$$

where ⊕ denotes Boolean summation, defined by 0 ⊕ 0 = 0, 0 ⊕ 1 = 1 ⊕ 0 = 1 ⊕ 1 = 1. The memory time of the cumulative adjacency matrix, defined by the parameter *n*, should be large enough so that for frozen systems an overwhelming majority of particles will visit the entire set of their neighbors during that time, but much shorter than the time it takes the particle to meet most of the particles in the system. I.e. the sum of terms in each row and column of the cumulative adjacency matrix should be much smaller than the total number of particles in the system. The former case without memory, described by the adjacency matrix at time *t*, is given by the case *n* = 0 in equation (5).

The Cumulative Mixing Rate (CMR), ${f}_{n}^{C}(t)$, is defined now in full analogy with equations (2–4), replacing the adjacency matrix by the cumulative adjacency matrix. (It is important to note that the Hamming distance between two consecutive cumulative adjacency matrices is not just the Hamming distance between the two extreme ordinary adjacency matrices appearing in the two consecutive cumulative adjacency matrices, which would have been the case if the summation on the right hand side of equation (5) would have been ordinary rather than Boolean). The parameter Δ*t* should be chosen as in equation (2) as a time increment, which is not too large yet large enough to have some sizeable effect in the adjacency matrix. It is interesting to obtain the mixing rate as a function of *n* which will tell us not only if the system is frozen or not but also on what scale it may be already considered frozen in the naïve sense.

Although our work is strongly motivated by study of granular systems, that when left alone freeze eventually, we prefer to demonstrate the new concept on an evolving, energy conserving many body system. The reason for that preference is that they provide the real acid test for the concept as follows from the discussion above. We consider a monodisperse system of 50,000 elastic frictionless spheres of radius = 0.5, interacting via a two body Hertzian interaction. Namely, the force that particle *i*, with center at **r**_*i*_ applies to particle *j* with center at **r**_*j*_ is given by^29–31^

6 $${{\bf{F}}}_{ij}=k\sqrt{R/2}\vartheta (\delta ){\delta }^{3/2}{{\bf{n}}}_{ij},$$

where *ϑ* is the Heaviside function, *δ* the linear overlap between spheres is given by $\delta =2R-|{{\bf{r}}}_{i}-{{\bf{r}}}_{j}|$ and ${{\bf{n}}}_{ij}$ is a unit vector pointing from the center of *i* to the center of *j*. The unit vector is perpendicular, of course, to the plane of contact. The strength of the interaction (elastic constant for normal contact) is given by *k* = 20000, and makes the spheres almost hard.

The Cumulative Mixing Rate (CMR) defined above is used to investigate the approach of agitated systems of such frictionless spheres to steady state. The simulations are performed using the LAMMPS Molecular Dynamics Simulator^32^, with a velocity-Verlet integrator^33^, in Lennard-Jones dimensionless units^34^. The spheres were placed according to the following protocol. The spheres were deposited within the top part of a box with dimensions: −20 ≤ *x*, *y* ≤ 20 and 0 ≤ *z* ≤ 200, given in L-J dimensionless units. The box has initially periodic boundary conditions in the *x*, *y* directions, and infinitely hard walls in the *z* direction. The *x*, *y* coordinates of the centers of the spheres are chosen randomly within the square, −19 < *x*, *y* < 19, while the *z* coordinate is chosen randomly between 50 and 199. The combined effect of gravity and a drag force is used to aggregate the spheres at the bottom of the box. The spheres are inserted in bunches, where each bunch is inserted only after the previous inserted particles have settled down. Gravity and drag force were then removed, and the box was compressed down in the z direction, to vary the volume fraction. A number of volume-fractions have been thus serially obtained and studied. For each volume fraction, the initial elastic energy was relaxed by evolving the compressed system, while adding a drag force on each of the spheres. Next, the drag force and was removed, and the boundary conditions in the *z* direction have been switched to periodic boundary conditions with the period being the distance between the hard walls, before switching the boundary conditions. The spheres were given initial random velocities, uniformly distributed, *v*_*x*_, *v*_*y*_, *v*_*z*_ ∈ *U* (−1, 1). The microscopic length that defines the range of adjacency is *l* = 1.6, which is 1.6 times the range of interaction between touching spheres. The adjacency data was recorded every *N*_*sim*_ = 2000 steps of the simulation of Δ*t*_sim_ = 0.0005. The number of steps between measurements was chosen so that ${v}_{rms}{N}_{sim}\Delta {t}_{sim}={v}_{rms}\Delta t\approx 1=2R$, is of the order of the spheres diameter and less than (but of order of) the range of adjacency, *l* = 1.6. To ensure consistency and conservation of energy, the kinetic energy was monitored. The following figures depict the dependence of the CMR on its order, *n*.

**Figure 2:**
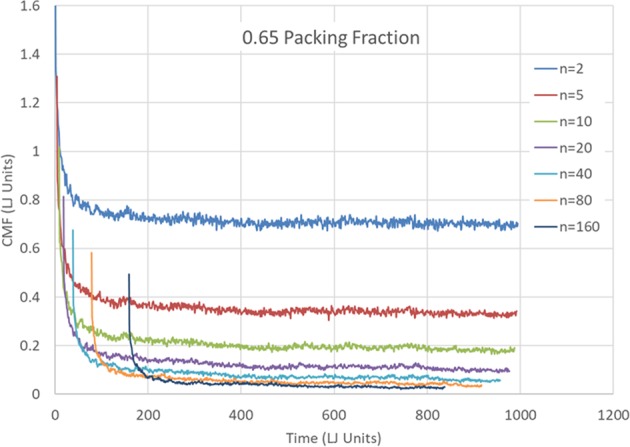
Mixing rate dependence on time and CMR order. The *n*’th order Cumulative Mixing Rate is depicted as a function of time for *n* = 2, 5, 10, 20, 40, 80 and 160, for a sample with a high-volume fraction of 0.65.

**Figure 3:**
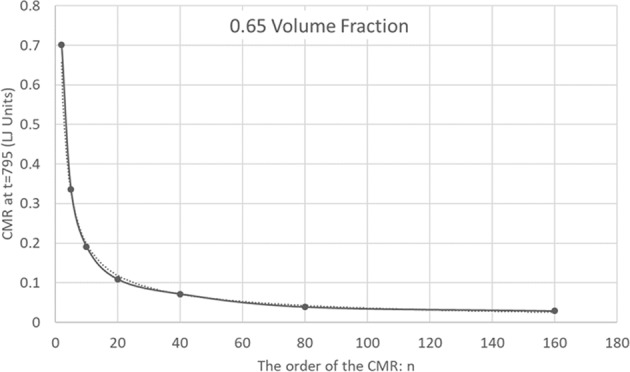
The *n*’th order Cumulative Mixing Rate as a function of *n*. The Cumulative Mixing Rate for the (high) 0.65 volume fraction at *t* = 795. The data points are derived from Fig. 2 by averaging the CMR between *t* = 790 and *t* = 800. Decaying Power law fitting suggests confinement of spheres and freezing.

In Fig. 2, the CMR, is plotted as a function of time for a high-volume fraction of 0.65 for orders *n* = 2, 5, 10, 20, 40, 80 and 160. As the order *n* increases, the CMR approaches zero. Whether the long-time limit is actually zero is unclear yet from our finite time simulation. Figure 3 depicts the CMR vs the order *n* for *η* = 0.65, and a fixed relatively long time, *t* = 795. To reduce fluctuations due to the finite number of spheres, the CMR was averaged over times between *t* = 790 and *t* = 800. The curve is fitted with a decaying power law, with an excellent agreement, ${f}_{n}^{C}(t=795)=1.099\cdot {n}^{-0.743}$. We have repeated the simulation with different (random) initial conditions and arrived in all cases to ${f}_{n}^{C}(t=795)=A\cdot {n}^{-\gamma }$, with *A* about 1.08 and *γ* about 0.75. This indicates that at least an overwhelming majority of particles are practically confined to cages consisting of their neighbors. This is not surprising since the volume fraction we chose is above the hard sphere crystallization value.

**Figure 4:**
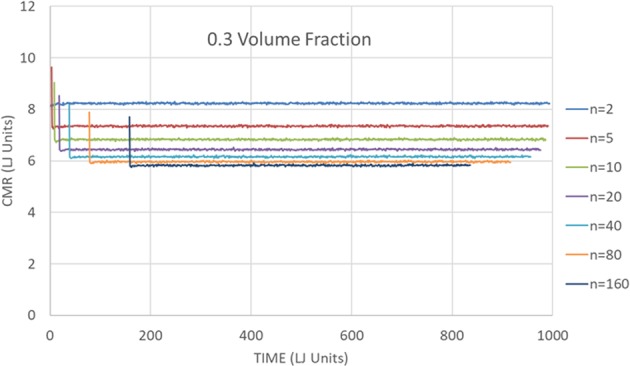
Mixing rate dependence on time and CMR order. The *n*’th order Cumulative Mixing Rate is depicted as a function of time for *n* = 2, 5, 10, 20, 40, 80 and 160, for a sample with a low volume fraction of 0.3.

**Figure 5:**
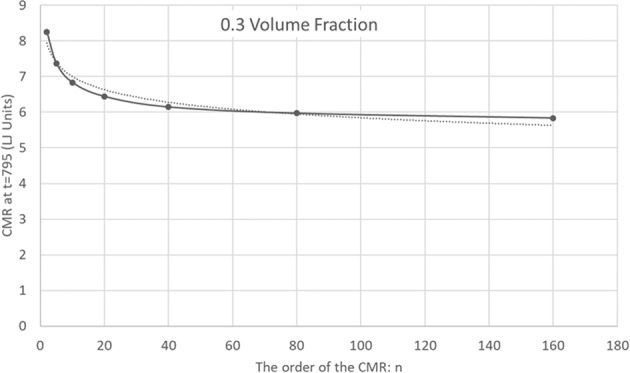
The *n*’th order Cumulative Mixing Rate as a function of *n*. The *n*’th order Cumulative Mixing Rate for the (low) *η* = 0.3 volume fraction, at *t* = 795. The data is derived from Fig. 4 by averaging the CMR over times between *t* = 790 and t = 800.

Figures 4 and 5, on the other hand, describe the CMR for a low volume fraction of 0.3. In Fig. 4 the CMR is depicted as a function of time. The CMR seems to settle to a constant value as a function of time after an initial drop. There is a decrease of the CMR with the order, *n*, but the drop is less pronounced for the higher orders. In Fig. 5 the CMR is presented as a function of *n* at *t* = 795. To reduce fluctuations, the average of CMR values for times between *t* = 790 and *t* = 800 is taken. The behavior is quite different from the one depicted in Fig. 3. The best power law fit, ${f}_{n}^{C}(t=795)=8.389{n}^{-0078}$, is considerably worse than the corresponding fit at *η* = 0.65. In fact, the larger *n* data points seem to flatten out more even than the weak dependence on *n* given by the best fit. This is expected, as long as *n* isn’t so large that each sphere explores the whole finite system.

**Figure 6:**
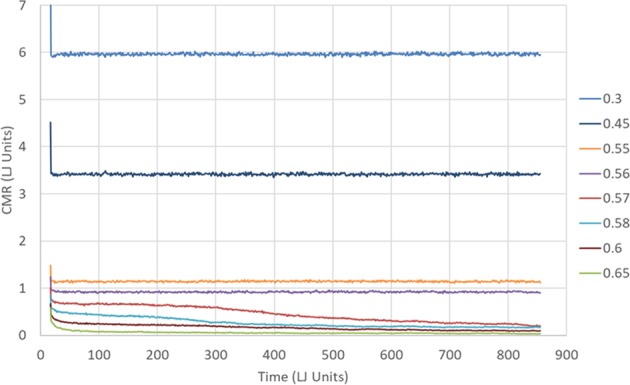
Cumulative Mixing Rate VS time and volume fraction. The Cumulative Mixing Rate of order 80 does not evolve with time for *η* ≤ 0.56. In contrast, the CMR decreases with time for higher volume fractions. The decay of the CMR seems to follow the freezing process.

The previous discussion suggests that *n* = 80 should be sufficient for our demonstration of freezing. Figure 6 shows the evolution of the CMR of order 80, ${f}_{80}^{C}(t)$, over time for the following volume fractions: 0.3, 0.45, 0.55, 0.56, 0.57, 0.58, 0.6 and 0.65, As the volume fraction is increased, the CMR decreases. This is expected due to the decrease in the mean free path. More interesting is the marked difference in the time evolution between low and high volume fractions. For all samples studied, there is a short time steep decrease in the CMR. Following that initial sharp decrease, the CMR at low volume fractions (*η* = 0.3, 0.45, 0.55 and 0.56) totally flattens out, while for the higher volume fractions a significant decrease of the CMR is visible over a long period of time. As the volume fraction increases the change happens faster. This may indicate freezing of the system around a volume fraction of 0.56. That value of *η* and the fact that our spheres are almost hard suggests, that the freezing we observe is actually crystallization rather than glass formation.

Thus, we have already demonstrated the applicability of mixing rate to identify the freezing transition. An interesting side issue is the nature of the frozen phase which will be discussed in the following. To determine the structure, we consider the number of neighbors, as defined by the adjacency matrix as a function of *l*. The motivation of doing that is that for a Bravais lattice we expect that the number of neighbors of a given site, ${\mathbb{N}}(l)$ plotted as a function of distance from that site, is just a series of steps with plateaus, corresponding to the total number of neighbors within a sphere of radius *l*. Even for lattices which are not Bravais lattices, in the sense that not all the lattice points are equivalent, the average number of neighbors as a function of *l* still consists of a series of steps. The reason is that this is true for each type of site. We cannot expect, of course, such a step structure for a lattice which is not frozen in the naïve sense but we certainly expect to see remnants of that step structure, if the freezing is really crystallization.

In the following we present the number of neighbors as a function of *l* for *η* = 0.56, 0.57 and 0.58. In each case we consider a single frame obtained at a late stage of the simulation corresponding to the longest times in Fig. 6.

**Figure 7:**
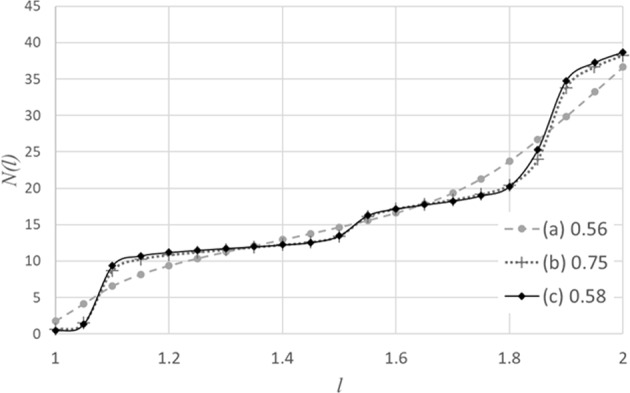
The average over all particles of the number of neighbors within a sphere of a radius *l*. (**a**) for volume fraction *η* = 0.56 (**b**) for *η* = 0.57 (**c**) for *η* = 0.58. Each figure corresponds to a single configuration obtained at a late stage of the simulation.

The difference between Fig. 7(a,b) is dramatic and clearly corresponds to the onset of freezing as seen in Fig. 6. Figure 7(b,c) suggest that the frozen structure is a crystal or very close to a crystal. The height of the nearest neighbor plateau, centered about 12 suggests that it is an fcc or hcp lattice. The second plateau is centered about 18, which is the sum of the number of nearest neighbors and next nearest neighbors in an fcc lattice.

In summary, we have introduced the Cumulative Mixing Rate (CMR), ${f}_{n}^{C}(t)$, that measures the freezing degree in classical many body systems. We have demonstrated the usefulness of the new concept by measuring it on an evolving Hamiltonian system of particles interacting via an almost hard sphere potential. The behavior of the CMR has been found to distinguish easily between the low density fluid phase and the high density frozen phase. Moreover, following the time evolution of the CMR enables actually to follow the process of freezing.

We chose a demonstration on a Hamiltonian system, because it is most difficult example on which the mixing rate could be tested. The concept can be applied also to systems with dissipation of energy, which freeze eventually but there the application should be even simpler than in the present case. In the present article we used the adjacency matrix not only to define the mixing rate but also to obtain the nature of the frozen state. We believe that the adjacency matrix (and related matrices) carry more interesting physical information, which we intend to study in the near future.
